# Source of Free Hydrochloric Acid in the Gastric Juice

**Published:** 1869-02

**Authors:** E. N. Horsford


### ARTICLE VIII.

### *Source of Free Hydrochloric Acid in the Gastric Juice.*

By Prof. E. N. Horsford.

The long disputed position of Prout, that the gastric juice contains free hydrochloric acid, was at length established by C. Schmidt, who, in an absolute quantitative analysis of the juice, found obout twice as much hydrochloric acid as was required to neutralize all the bases present.

The prolonged discussion of this subject, now since 1823, has brought to light, through the researches of Lassaigne, Claude Bernard, Schwann, and numerous others, the unmistakable evidence of the presence of lactic acid and of acid phosphates in the gastric juice, which latter might or might not be due to the presence of lactic or hydrochloric acid.

A point of special interest to the chemist and physiologist still remained, and was this:

*How could free hydrochloric acid be secreted from the blood, which is an alkaline fluid?*

The blood, freshly drawn, consists of a fluid (the plasma), in which there are swimming myriads of exceedingly minute, irregularly spheroidal bodies (the corpuscles). The plasma consists of two bodies—one of which, the fibrin, spontaneously separates from the other, the serum. The corpuscles are little sacs of delicate animal membrane, enclosing a fluid. This fluid has an acid reaction, and its ash contains a monobasic alkaline phosphate. The fibrin of the plasma contains, according to Virchow, a glycero-phosphate of lime, though the plasma, as a whole, has an alkaline reaction, and contains, in its ash, a great measure (eleven per cent.) of chloride of sodium.

The moist corpuscles constitute about one-half of the blood.

In healthy digestion, the blood-vessels of the stomach are engorged. Engorgement is the equivalent of obstruction. This must occur in the capillaries, where the diameter of the blood-vessels is least.

The plasma, because of its fluidity, must move more freely than the corpuscles. The proportion of the corpuscles in the capillaries will be thereby relatively increased. Under the pressure that follows, the fluid contents of the corpuscles will pass through their membranous walls, and, mingling with the relatively lessened plasma, pass on through the walls of the capillaries. This mixture will therefore contain acid phosphates and chloride of sodium.

The mucous membrane of the stomach presents, on its inner surface, the mouths of numerous microscopic tubes, which like stockings, are sometimes single blind sacs, or, like gloves, terminate in several blind sacs, like the glove-fingers. In the bottoms of these tubes, and along their sides, are several closed spherical sacs, containing other lesser sacs, and fluid within. The tubes, as a whole, dip down into the spongy tissue that underlies the mucous coat, where they are surrounded by the fluid, poured from the network of nutritive capillaries, which fluid, as remarked above, contains acid phosphates and chlorides.

Now, by pressure and osmosis, a portion of this fluid will pass through the walls of the gastric tubes, and the question is:—

*Whether the fluid that goes through will contain free hydrochloric acid?*

The experiments I have made are conclusive on the principal point.

By employing acid phosphate of lime and common salt, I had this advantage, that as increased acidity on the one hand is a just inference from increased alkalinity on the other, and as increased alkalinity would be shown by the precipitation of phosphate of lime—a visible white powder—I could determine the qualitative fact without the difficulties and delay attending on accurate quantitative analysis of the solution before and after the experiments, on both sides of the membrane.

I employed an acid phosphate of lime of specific gravity 1.117—of a constitution of 3 ($CaO$ $PO_5$)+2 $PO_5$ —with an amount of phosphate of peroxide of iron present, as one to twenty-eight of the acid phosphate of lime. The other solutions employed were the ordinary laboratory reagents.

On adding ammonia, in small quantities, to the solution of acid phosphate, with alternate agitation, it required, as might be inferred, several repetitions before the peroxide, with its phosphoric acid, became a permanent precipitate, and still several more before the precipitate of phosphate of lime became permanent.

In my earlier experiments, in which I employed parchment paper, I was embarrassed with the presence of sulphate of lime in the precipitated powder, so that what was at first supposed to be phosphates of lime and iron, was found to be in part, sulphate of lime. This sulphate was due to imperfectly-washed parchment-paper, which still contained sulphuric acid. This difficulty overcome, the experiments were made with parchment-paper, prepared from German and Swedish filter-paper, as well as with gold-beater's skin (animal membrane).

I employed the acid phosphate of the formula above, with (each by itself) chloride of sodium, chloride of ammonium, chloride of potassium, chloride of magnesium, chloride of calcium and acetate of potassa.

With all of these, there was obtained the same kind of evidence of increased acidity on one side, and of increased alkalinity on the other—to wit, the powder thrown down from the mixture of acid phosphates and chloride. What successive additions of ammonia had been required to effect, had been accomplished by dialysis.

The same effect took place from a mixture of acid phosphate of soda and chloride of calcium.

It follows from the above, if these experiments fairly represent the case, and from the known composition of the blood, its condition in the walls of the stomach, and the structure of the gastric tubules, that free or uncombined hydrochloric acid must find its way into the bottoms of the gastric tubules, and thence into the cavity of the stomach

It may be urged that I should show that the acid phosphate, pressed from the corpuscles, more than neutralizes the alkalinity of the plasma present. In reply, it may be said that I present a condition of things in which there is the *kind* of physical change required *going on*—namely, relative augmentation of the corpuscles, under pressure, the concomitant of engorgement.* Its *degree* must be inferred from the effects on the secretions, which I have endeavored to point out, by conducting an experiment under what I conceive to be essentially like conditions, and obtaining the result due to identical conditions.

The secretion of hydrochloric acid is, of course, mixed with acid phosphates and alkaline chlorides.

That such a result, as I have arrived at, would follow experiment, might have been predicted from Graham's researches in dialysis. Phosphates of lime and soda are colloidal, relatively, to more crystalloidal hydrochloric acid. Graham found that bisulphate of potassa, by dialysis, was resolved into two salts, or mixtures, of greater and lesser acidity than the original bisulphate. So he found that acetate of peroxide of iron was resolved by dialysis into hydrated peroxide of iron, and free acetic acid. It is possible, and probable, that the albuminoid bodies present take part in determining the contrast between colloid and crystalloid bodies. Graham found that, by dialysis, he could separate free hydrochloric acid from the gastric juice, thrown up by vomiting.

It may be further objected that anatomists are not agreed as to the structure of the corpuscles. But it will be seen that there is no more required than may be regarded as established. The corpuscles act in many particulars, if not all, as if they were membranous sacs, more or less distended with fluid. They may be swollen by immersion in a thinner (less colloid) fluid, and reduced by immersion in a more colloid fluid; that is, they are susceptible of endosmosis and

* I employ the word "engorgement" simply as implying the condition of increased pressure in the capillaries. With constant elasticity of the walls of the capillaries, increased pressure would accompany increased flow of blood.

exosmosis, as membranous sacs would be. In their ordinary condition, as seen under the microscope, they present the appearance of collapsed spherical or oval sacs or cells. They appear as double concave disks. In swelling (by endosmosis) the lowest part of each concaivty is the last to take on the spherical contour, just as they woul do if they were membranous sacs. The corpuscles sometimes so collapse (by exosmosis) that one-half of the hollow sphere is reversed while the other half retains its form unchanged, the former setting like a cup in the latter—a conformation inconceivable on the theory of homogeneity of the corpuscles as a whole. Crystallizable substances may be extracted from the corpuscles by pressure and by endosmosis. They must have been in solution in order to crystallization, and solution involves a fluid.

The liquid expressed from the corpuscles has an acid reaction aud contains an organic acid and acid phosphates. It contains among other bodies the hæmatoidin of Virchow. The ash of these crystals consists almost wholly of metaphosphates* which point directly to tribasic phosphoric acid in solution, combined with one atom of fixed base, which is inconceivable unless separated by membrane from the plasma, which is always alkaline.

In fine, whatever other peculiarities the blood-corpuscles may possess, they have the requisites for furnishing acid phosphates in solution, under pressure such as must attend engorgement of the capillaries in the walls of the stomach.

Let us glance at what takes place in all probability as the acid fluid enters the gastric tubules. Here are sacs containing fluid at the bottom and along the sides of the tubules. They are surrounded by a mixture of hydrochloric acid, acid salts, neutral salts, and albuminoid bodies. Dialysis must be repeated and a stronger acid solution pass into the sacs. The sacs, swelling by endosmosis and corroded by the acid,

* The ether extract of the blood-corpuscles yields, according to Schwann, an ash containing acid phosphate of soda. Owen Reese and Berzelius maintained the existence of oleo-phosphoric acid in the corpuscles. Andral [Berzelius's *Jahrs-Bericht* 1547–'8, p. 894] places the contents of the corpuscles among the acid fluids of the body.

must at length burst, and the liquid contents, together with the disintegrated and partially digested membrane of the sacs, pass out to the stomach to constitute the gastric juice —the free hydrochloric acid, acid phosphates and chlorides, and the albuminoid bodies and disintegrated tissue (the pepsin ?) to act in the liquefaction of food.—*New York Medical Journal.*

---

